# Association of Diet, Physical Activity Guidelines and Cardiometabolic Risk Markers in Children

**DOI:** 10.3390/nu13092954

**Published:** 2021-08-25

**Authors:** Mercedes Gil-Campos, Alexandra Pérez-Ferreirós, Francisco Jesús Llorente-Cantarero, Augusto Anguita-Ruiz, Juan José Bedoya-Carpente, Anton Kalén, Luis A. Moreno, Gloria Bueno, Ángel Gil, Concepción M. Aguilera, Rosaura Leis

**Affiliations:** 1 CIBEROBN, (Physiopathology of Obesity and Nutrition) Institute of Health Carlos III (ISCIII), 28029 Madrid, Spain; mercedes_gil_campos@yahoo.es (M.G.-C.); llorentefj@yahoo.es (F.J.L.-C.); augustoanguitaruiz@gmail.com (A.A.-R.); lmoreno@unizar.es (L.A.M.); agil@ugr.es (Á.G.); caguiler@ugr.es (C.M.A.); 2 Metabolism and Investigation Unit, Reina Sofia University Hospital, Maimónides Institute of Biomedicine Research of Córdoba (IMIBIC), University of Córdoba, 14071 Córdoba, Spain; 3 Unit of Investigation in Human Nutrition, Growth and Development of Galicia (GALINUT), University of Santiago de Compostela (USC), 15706 Santiago de Compostela, Spain; alexandra15pf@gmail.com (A.P.-F.); xanthebusker@gmail.com (J.J.B.-C.); anton.kalen@gmail.com (A.K.); 4 Department of Specific Didactics, Faculty of Education, University of Córdoba, 14004 Córdoba, Spain; 5 Center of Biomedical Research, Department of Biochemistry and Molecular Biology II, Institute of Nutrition and Food Technology “José Mataix”, University of Granada, 18016 Granada, Spain; 6 Biosanitary Research Institute (IBS), 18014 Granada, Spain; 7 GENUD Research Group, Institute of Sanitary Research of Aragón (IIS Aragón), University of Zaragoza, 50009 Zaragoza, Spain; 8 Agri-Food Institute of Aragon (IA2), 50009 Zaragoza, Spain; 9 Unit of Pediatric Endocrinology, University Clinical Hospital Lozano Blesa, 50009 Zaragoza, Spain; 10 Unit of Pediatric Gastroenterology, Hepatology and Nutrition, Pediatric Service, University Clinical Hospital of Santiago (CHUS), 15706 Santiago de Compostela, Spain; 11 Pediatric Nutrition Research Group, Institute of Sanitary Research of Santiago de Compostela (IDIS), CHUS–USC, 15706 Santiago de Compostela, Spain

**Keywords:** abdominal adiposity, cardiovascular diseases, child, child nutrition sciences, diet, exercise, metabolic syndrome, metabolism, obesity, pediatric obesity

## Abstract

The aim was to identify different dietary and physical activity (PA) patterns in 5- to 14-year-old children with a high prevalence of overweight and obesity using cluster analysis based on their adherence to the Spanish Society of Community Nutrition dietary guidelines and levels of PA, and to determine their associations with age, sex, body composition, and cardiometabolic risk markers. In 549 children, hierarchical cluster analysis was used to identify subgroups with similar adherence to dietary recommendations and level of PA. Three clusters were identified: Cluster 1, with the lowest level of vigorous PA and adherence to dietary recommendations; Cluster 2, with the lowest levels of moderate and vigorous PA and the highest adherence to dietary recommendations; and Cluster 3, with the highest level of PA, especially vigorous PA and a medium level adherence to dietary recommendations. Cluster 3 had lower total body fat and higher lean body mass percentages than Cluster 2. Cluster 2 had lower high-density lipoprotein cholesterol and higher low-density lipoprotein cholesterol levels than Cluster 1. The results from our study suggest that it is important to consider adherence to PA recommendations together with adherence to dietary guidelines to understand patterns of obesogenic habits in pediatric populations with high prevalence of overweight and obesity.

## 1. Introduction

Obesity is a major health concern in developed societies and with the rising prevalence of childhood obesity, the problem is expected to increase further in the future [1,2]. Childhood obesity affects child’s quality of life and is associated with a wide variety of disorders, such as metabolic syndrome (MetS) [3,4]. Moreover, it also has cardiometabolic implications later in life [5].

Dietary habits are an important factor to consider in preventing obesity [6,7]. Therefore, scientific organizations have established healthy dietary guidelines to prevent them [8]. However, adherence to the healthy Mediterranean diet has been gradually decreasing over the last few decades in the Mediterranean region, especially in children [9,10].

Additionally, regular physical activity (PA) plays an important role in preventing obesity and related morbidities, as it leads to reduced body fat mass and increased cardiorespiratory fitness [11,12]. The World Health Organization (WHO) has, therefore, established guidelines for PA for children and adolescents [13].

It is important to consider both dietary and PA patterns and how they interact to identify obesogenic habits. Childhood is a critical period for establishing a healthy lifestyle which reduces the risk of obesity and related morbidities in childhood and adulthood [1,14,15]. For example, the HELENA study, with a European, and the EsNuPi study, with a Spanish general population of children and adolescents relate clusters of lifestyle patterns to sex, age, and socioeconomic factors [16,17]. However, few studies has focused on clusters of lifestyle patterns in populations of children with a high prevalence of overweight and obesity, and how they might relate to cardiometabolic risk [18].

With the rising prevalence of childhood obesity, as well as declining adherence to dietary and PA guidelines, it is becoming increasingly important to study the lifestyle patterns and how they relate to body composition and cardiometabolic risk markers in a population with a higher prevalence of overweight and obesity than the current general population [17]. Therefore, the present study aimed to identify different dietary and PA patterns in Spanish 5- to 14-year-old children visiting nutrition and pediatric endocrinology units using cluster analysis based on their adherence to the recommendations and determine associations between the identified lifestyle patterns and age, sex, body composition, and cardiometabolic risk markers.

## 2. Materials and Methods

The present study is a component of the GENOBOX study, an observational, cross-sectional, multicenter study carried out in Spain between 2012 and 2015 [2]. In the GENOBOX study, a total of 813 children from the nutrition and pediatric endocrinology units of 3 third-tier hospitals in Spain were included [2]. In the present study, a subsample of 549 children was selected based on the inclusion criteria: 5–14-year-old children of both sexes who did not suffer from any chronic pathology other than obesity and who had completed dietary and PA questionnaires (Figure S1). Individuals who had received any pharmacological or dietary treatment that could interfere with the results in the year prior to the start of the study were excluded.

The following parameters were evaluated: PA intensity and time, dietary habits, body composition, and blood cardiometabolic risk markers.

The study was designed following the ethical principles for human research of the Declaration of Helsinki of 1964, last revised in 2013, in Fortaleza, Brazil. It was approved by the ethics committees and research committees of the participant centers: 2011/198 and 12/2010. All the participants and their families were informed about the study and voluntarily agreed to participate. For every child, informed consent from their parents or legal guardian was obtained, and consent from participants over 12 years old was obtained.

### 2.1. Physical Activity

The Spanish version of the International Physical Activity Questionnaire (IPAQ) was used to collect information about the participants time engaged in PA at different intensities [19]. Based on the results, we recorded if the participants met the WHO recommendations for PA in two variables: moderate-to-vigorous PA (MVPA) adherence and full PA adherence [13]. MVPA adherence refers to whether the participant met the recommendations for 60 min of daily MVPA or not. Full PA adherence refers to whether the participant met the recommendations for 60 min of daily MVPA and three days of vigorous PA or not. Further, another two variables were registered: moderate PA (MPA) time and vigorous PA (VPA) time, measured as minutes per week, and used in the cluster analysis.

### 2.2. Dietary Habits

Dietary habits were assessed by using a validated and reliable food frequency questionnaire [20,21,22]. Response options displayed from left to right were as follows: never or hardly ever; 1–3 times per month; 1, 2–4 or 5–6 times per week; and 1, 2–3, 4–6 or >6 times per day.

Participants were given a score of 0 to 8 points for each type of food based on the degree of adherence to the recommendations of the Spanish Society of Community Nutrition (SENC) (Table 1) [8].

The food types were categorized into two groups: foods to be promoted and foods to be avoided. Additionally, the average adherence score was calculated separately for foods to be promoted and foods to be avoided. Detailed description of how the adherence was scored from the validated questionnaire for each type of food can be found in Table S1.

### 2.3. Body Composition

Body composition was determined with two parameters, body mass index (BMI), and the results of dual-energy X-ray absorptiometry (DEXA). Participants were weighed and sized as reported elsewhere [2]. BMI (weight/height^2^) was stratified into three groups according to the international standard of Cole et al. [23] LunarEncore^®^ DEXA (GE Healthcare; Chicago, IL, USA) allowed us to assess fatty tissue and lean boneless tissue. Fat-free mass index (FFMI) and fat mass index (FMI) were calculated according to Kyle et al. [24].

### 2.4. Blood Cardiometabolic Risk Markers

Blood samples for the evaluation of cardiometabolic markers were obtained through venipuncture performed after at least 12 h of fasting and within the previous 6 h of sampling. Intense PA was not performed within the hour before sampling. The samples collected in EDTA tubes were centrifuged and immediately stored at −80 °C. Systolic (SBP) and diastolic (DBP) blood pressure, and fasting glucose, insulin, total cholesterol, triacylglycerols, high-density lipoprotein (HDL-c) and low-density lipoprotein (LDL-c) cholesterol were measured using standardized techniques, as previously reported [2]. Homeostatic model assessment (HOMA-IR) was calculated using the following formula: (glucose (mmol) × insulin (μU/mL))/22.5. MetS was classified according to Olza et al. [4].

### 2.5. Statistical Analysis

All continuous variables were non-normally distributed. Therefore, median and interquartile range (IQR) was reported and the rank-biserial correlation (rrb) with 95% confidence interval (CI) was used to compare groups. The rrb ranged from −1 to 1 and indicated the probability that a participant from the second group has a higher value that a participant from the first group. For categorial variables, *N* (%) was reported, together with the difference in percentage between groups with 95% CI. If both limits of the 95% CI have the same sign (i.e., both positive or both negative), the difference between groups is significant (*p* < 0.05).

Clusters were defined based on standardized values of weekly minutes of moderate PA and vigorous PA, as well as scale of adherence to recommendations of foods to be promoted and foods to be avoided. Agglomerative hierarchical clustering with Ward’s method and Euclidian distances was carried out using the “agnes” function from the R package “cluster”. After visual inspection, we determined the optimal number of clusters to be k = 3. Full details of the cluster procedure can be seen in Supplementary Material S1.

All statistical analyses were performed in R 4.0.3.

## 3. Results

A total of 549 children were included in the study; 54% were females and excess weight was observed in 73.1%. Table 2 presents general characteristics of the full sample and comparisons between sexes; 14.5% presented MetS, 74.6% of the participants did not meet the MVPA recommendations, and 93.1% did not meet the full PA recommendations. Participants carried out a median of 180 min/week of moderate and 0 min/week of VPA. Males spent significantly more min per week performing both MPA and VPA than female participants. No significant differences in the consumption of foods to be promoted or to be avoided between sexes were observed, although female participants presented better scores for both variables.

**Figure 1:**
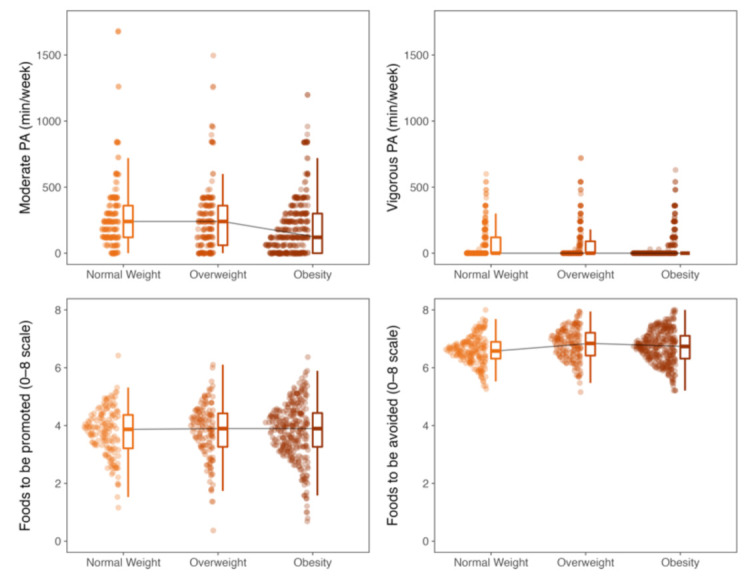
Amount of physical activity (PA) and dietary adherence by BMI categories.

MPA and VPA times as well as adherence to nutritional recommendations in the different BMI categories can be seen in Figure 1, and all comparisons are presented in Table S2. Participants with obesity spent significantly less time performing MPA (120, IQR = 0–300 min/week) than those with overweight (210, IQR = 63–60 min/week) and normal weight (240, IQR = 120–360 min/week). Moreover, participants with normal weight had significantly poorer adherence to foods to be avoided (6.58, IQR = 6.26–6.84) than both overweight (6.84, IQR = 6.39–7.13) and obesity (6.74, IQR = 6.32–7.11); no significant differences were found in adherence to the consumption of foods to be promoted.

Of the participants with obesity, 30.4% presented MetS. There were no significant differences between participants with and without MetS in PA and nutritional adherence variables (Table S3).

Comparisons between participants who met, and did not meet, MVPA recommendations are presented in Table S4. Participants who met MVPA recommendations engaged more time in both MPA (420, IQR = 360–600 vs. 120, IQR = 0–240 min/week) and VPA (90, IQR = 0–255 vs. 0, IQR = 0–0 min/week) than participants who did not. They had also a significantly lower FMI (3.89, IQR = 3.26–4.47 vs. 3.95, IQR = 3.26–4.42 kg/m^2^) and higher FFMI (6.74, IQR = 6.34–7.11 vs. 6.68, IQR = 6.32–7.00 kg/m^2^).

Comparisons between participants who met, and did not meet, full PA recommendations are presented in Table S5. Participants who met the full PA recommendations had a significantly higher FFMI (14.6, IQR = 13.3–16.2 vs. 13.2, IQR = 12.2–14.6 kg/m^2^) than participants who did not.

### Clusters of Adherence to Dietary and Physical Activity Recommendations

**Figure 2:**
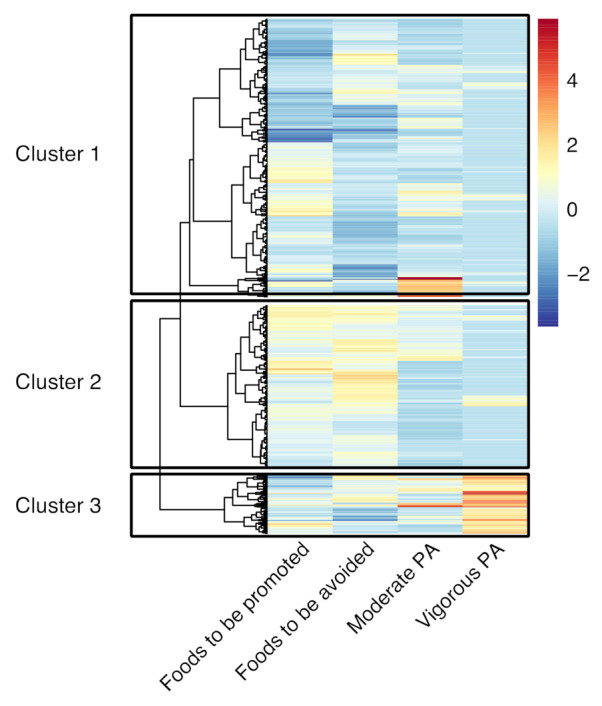
Heatmap of participants’ adherence to dietary recommendations and levels of physical activity (PA). The dendrogram shows the proximity between participants, which are divided into three clusters. The colors indicate standardized levels of adherence to dietary recommendations and the levels of PA.

**Figure 3:**
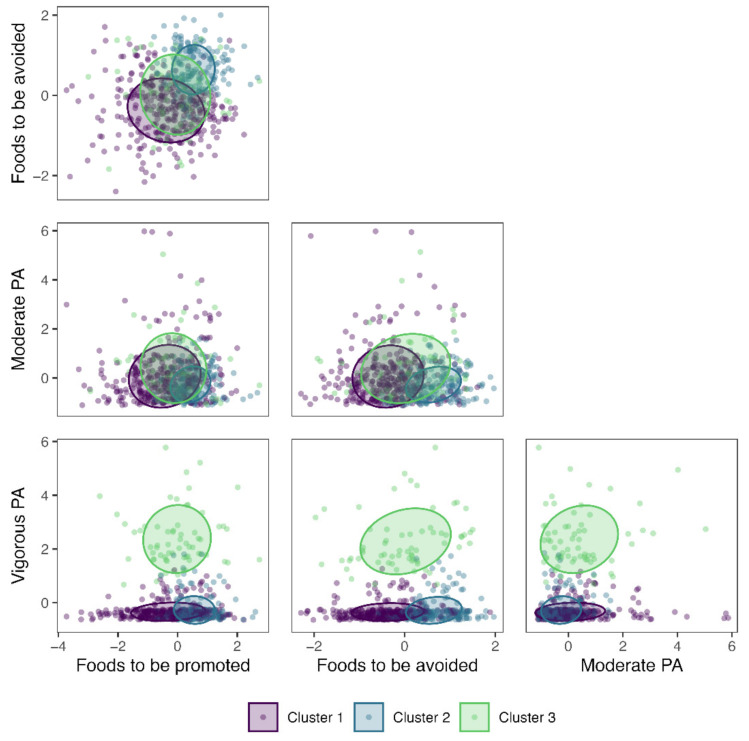
Clustered standardized scores of adherence to dietary recommendations and levels of physical activity (PA). Ellipses represent 50% confidence levels.

The participants were clustered based on standardized measures of MPA and VPA time, consumption of foods to be promoted, and consumption of foods to be avoided (Figure 2). Higher values of the variables indicated better adherence to the nutritional recommendations and a higher level of PA. Cluster 1 was characterized by the lowest adherence to both foods to be promoted and to be avoided, a medium level of MPA, and the lowest level of VPA (Figure 3). Cluster 2 was characterized by the highest adherence to both foods to be promoted and to be avoided and the lowest levels of both MPA and VPA. Cluster 3 exhibited medium adherence to both type of foods and the highest levels of MPA and VPA. The consumption of foods to be promoted and the consumption of foods to be avoided differed between all three clusters, while MPA differed Cluster 2 from the other two clusters, and VPA differed Cluster 3 from the other two clusters (Table 3). Overall, VPA (Kruskal–Wallis H = 296.23) and the consumption of foods to be avoided (Kruskal–Wallis H = 201.00) were substantially more influential in clustering participants than the consumption of foods to be promoted (Kruskal–Wallis H = 93.70) and MPA (Kruskal–Wallis H = 20.75).

MVPA adherence refers to the number of participants who met the recommendations for 60 min/day of MVPA. Full PA adherence refers to the number of participants who met the recommendations for 60 min/day of MVPA and three days of vigorous PA. Promoted and avoided foods scales correspond to the degree of adherence to food consumption recommendations.

Cluster characteristics, dietary adherence, PA, body mass, and cardiometabolic markers are shown in Table 3. The majority of the participants were included in Cluster 1 (55.7%). Approximately 12% of the participants, with an overrepresentation of males, were included in Cluster 3. Cluster 3 had a higher proportion of participants with normal weight than the other clusters.

## 4. Discussion

The main findings of the present work were that we identified three clusters of children based on their adherence to dietary and PA recommendations, one cluster with unhealthy behaviors and two clusters with mixed behaviors. The cluster with highest PA, especially vigorous, showed better body composition, while the cluster with better dietary adherence but insufficient PA showed the worst lipid profile.

The prevalence of obesity and MetS was higher than previously reported [4]. These results can be explained by the fact that we recruited participants from endocrinology and nutrition units, in which more than 70% of the patients have overweight or obesity.

Only 25.4% of the participants followed the WHO recommendations regarding MVPA [13], higher than the 18.6% adherence rate reported in 479,674 children and adolescents from 32 countries [25]. We found that boys spent significantly more time performing both MPA and VPA than girls and had better adherence to the PA recommendations. The sex differences are in line with Kalman et al., who found that 23% of school-aged boys but only 14% of girls adhered to MVPA recommendations [25].

The participants showed a relatively high adherence to the recommendations of foods to be avoided, while the recommendations of foods to be promoted were followed to a lesser degree. When comparing participants categorized by BMI, we found no significant differences in adherence to foods to be promoted. In contrast, the participants with normal weight had the lowest adherence to foods to be avoided. A recent meta-analysis found an association between unhealthy dietary patterns and health measures such as body composition and cardiometabolic blood markers, but no association for healthy dietary patterns [26]. In contrast, children with obesity presented a lower adherence to MVPA and full PA recommendations in our study. This might suggest the important role that PA play in the changes in body mass in our sample of children with high prevalence of overweight and obesity. In line with our results, dietary patterns tend to be less important than PA and sedentary behaviors when determining obesogenic patterns in children [18].

Participants who adhered to the full PA recommendations did not differ significantly in total and abdominal fat mass percentage from those who did not, in contrast with earlier findings [27]. This difference could be due to the small number of participants who adhered to the full PA recommendations. However, we found differences in the percentages of total fat and lean mass between participants who met and did not meet the MVPA recommendations.

In relation to the clustering approach, 55.7% children in the present study were classified in Cluster 1, with the unhealthiest behaviors regarding both diet and vigorous PA. This is a notably higher percentage than that in a previous study in European adolescents [17]. In general, previous studies have found that most children and adolescents have complex patterns of mixed healthy and unhealthy PA, dietary, and sedentary behaviors, as observed in clusters 2 and 3 in the present study [18,28]. Only 11.8% of the participants were classified in Cluster 3, with the highest level of PA.

Male participants were overrepresented in Cluster 3, which performed more PA than the other clusters. Female participants were overrepresented in Cluster 2, which had healthy dietary patterns but the lowest level of PA. Previous studies have consistently found similarly high proportions of boys in high-PA clusters and girls in low-PA clusters [17,18]. The higher percentage of total fat in Cluster 2 is likely the result of the higher fat percentage in female participants. However, while Cluster 2 had the lowest abdominal fat percentage, females had a higher percentage of abdominal fat than males. 

Cluster 3 had lower proportions of overweight and obese participants, a lower total fat mass percentage and a higher lean mass percentage than the other two clusters. This finding can be expected since Cluster 3 had an overrepresentation of males and was characterized by the highest level of PA. The majority of previous studies found an association between lifestyle patterns characterized by a high level of PA and low BMI [18,29].

Cluster 3 also had the highest levels of HDL-c, while Cluster 2 had the highest levels of LDL-c. Nevertheless, all groups had HDL-c and LDL-c levels within the normal ranges. Although certain dietary patterns—such as high omega-3 consumption and the Mediterranean diet—have been linked to higher HDL-c levels, the effects are relatively modest, and there is evidence that PA has a greater impact on lipid profiles than omega-3 consumption [30,31]. HOMA-IR is also affected by PA and body lean mass [32,33]. This is in line with our finding that the higher-PA cluster seemed to have lower insulin resistance and better HDL-c levels than the lower-PA clusters. However, we found that the prevalence of MetS was similar among the three clusters, which might indicate that neither high levels of PA nor dietary adherence alone would be sufficient to avoid MetS. It is possible that both high levels of PA and high adherence to a healthy diet are needed to reduce the risk of MetS.

The cluster analysis did not suggest any profile of participants characterized by both healthy dietary and healthy PA patterns. It classified the lifestyle of approximately 56% of the participants as unhealthy, with poor dietary and PA habits, while the remaining 44% had a mixed healthy and unhealthy lifestyle. This might be explained by the fact that the sample was recruited in the nutrition and pediatric endocrinology units of three third-tier hospitals in Spain, and a high percentage of children had high adiposity. However, recent studies such as EsNuPi, showed a poor quality of the diet in a general pediatric Spanish population [34]. In a similar way, other studies found that few children achieve the recommended level for health of moderate PA [35].

Analysis of body mass and cardiometabolic markers, especially the comparison of the two clusters with mixed behaviors, might suggest that a healthy dietary pattern alone might be insufficient to stay healthy and that a sufficient level of PA is needed. We must keep in mind that the sample with high prevalence of overweight and obesity might explain the higher levels of fat mass and cardiometabolic risk markers. However, only participants without any chronic pathology other than obesity nor any pharmacological or dietary treatment, were included.

The interpretation of the present study results is fundamentally limited because it was an observational study with nonrandom sampling, and lifestyles, diet and PA were collected using self-reported questionnaires.

Future controlled intervention trials related to the adherence of PA and dietary recommendations should be conducted. These studies could also incorporate other possible risk parameters, such as sleep and mental health status.

## 5. Conclusions

In the present study, we identified three clusters associated with adherence to dietary and PA recommendations in a clinical cohort of Spanish children. Cluster 1 was characterized by the lowest adherence to foods to be promoted and to be avoided, a medium level of moderate PA, and a low level of vigorous PA; Cluster 2 was characterized by the highest adherence to foods to be promoted and to be avoided but the lowest levels of moderate and vigorous PA; and Cluster 3 was characterized by the highest levels of moderate and especially vigorous PA and a medium adherence to dietary recommendations. We did not identify any cluster of children who had both high levels of PA and healthy dietary patterns. The cluster with high PA but medium dietary adherence had a lower fat mass, higher lean mass, higher HDL-c, and a tendency toward less insulin resistance than the other two clusters.

The results from our study suggest that it is important to consider adherence to PA recommendations together with adherence to dietary guidelines to understand patterns of obesogenic habits in pediatric populations with high prevalence of overweight and obesity.

## Figures and Tables

**Table 1:** SENC food consumption recommendations [8].

Foods to Be Promoted	Foods to Be Avoided
Dairy	Sugar, jam, cocoa
Fruits	Sweet snacks, salty sacks
Vegetables	Soft drinks
Whole grains and potatoes	Precooked food
Olive oil ^1^	Margarine, butter, sunflower oil
Lean meats ^2^	Sausages
Eggs	
Fish ^3^	
Legumes	

SENC (Spanish Society of Community Nutrition). ^1^ Raw or cooked. ^2^ Lean pork, beef, chicken, or turkey. ^3^ Blue and white fish.

**Table 2:** Characteristics of all the variables for the total sample and by sex.

		Sex		ES	95% CI
	Total	Female	Male		
*N*	549	296 (53.9%)	253 (46.1%)		
Age (years)	10.52 (8.67–12.30)	10.50 (8.42–12.33)	10.53 (8.75–12.30)	−0.01	−0.11–0.09
BMI category					
Normal weight	143 (26.0%)	67 (22.6%)	76 (30.0%)	−7.4%	−15.0–0.4%
Overweight	131 (23.9%)	77 (26.0%)	54 (21.3%)	4.7%	−2.8–12.0%
Obesity	275 (50.1%)	152 (51.4%)	123 (48.6%)	2.7%	−6.0–11.0%
Physical activity					
MVPA adherence	139 (25.4%)	66 (22.4%)	73 (28.9%)	−6.5%	−14.0–1.2%
Full PA adherence	38 (6.9%)	16 (5.4%)	22 (8.7%)	−3.3%	−8.0–1.4%
Moderate PA (min/week)	180 (60–300)	120 (60–300)	180 (60–360)	−0.11	−0.21–−0.02
Vigorous PA (min/week)	0 (0–0)	0 (0–0)	0 (0–60)	−0.10	−0.20–0.00
Dietary adherence					
Promoted foods (0–8 scale)	3.89 (3.26–4.42)	3.89 (3.32–4.39)	3.84 (3.16–4.42)	0.04	−0.05–0.14
Avoided foods (0–8 scale)	6.68 (6.32–7.05)	6.74 (6.37–7.05)	6.68 (6.32–7.05)	0.03	−0.07–0.13
Body mass					
Total fat mass (%)	39.5 (31.8–45.0)	41.0 (35.7–46.8)	36.9 (28.1–43.1)	0.28	0.16–0.38
Abdominal fat (%)	8.9 (8.0–10.1)	9.2 (8.2–10.4)	8.6 (7.8–9.9)	0.18	0.04–0.31
Total lean mass (%)	59.3 (53.3–67.1)	57.6 (51.2–62.9)	61.9 (55.3–70.4)	−0.29	−0.39–−0.18
FFMI (kg/m^2^)	13.3 (12.3–14.8)	13.1 (12.0–14.5)	13.6 (12.5–14.9)	−0.15	−0.26–−0.03
FMI (kg/m^2^)	0.9 (0.7–1.2)	1.0 (0.7–1.2)	0.8 (0.5–1.1)	0.22	0.10–0.33
Cardiometabolic Risk Markers					
Triacylglycerols (mg/dL)	62 (46–84)	64 (52–90)	55 (41–79)	0.20	0.11–0.30
Cholesterol (mg/dL)	162 (144–181)	163 (142–180)	161 (144–182)	−0.01	−0.11–0.08
HDL−c (mg/dL)	49 (41–60)	48 (40–57)	51 (43–62)	−0.14	−0.23–−0.04
LDL−c (mg/dL)	94 (79–109)	93 (79–109)	94 (80–108)	−0.02	−0.12–0.08
Insulin (mU I/L)	10.6 (6.1–15.7)	12.3 (7.8–17.2)	8.9 (4.7–13.7)	0.26	0.17–0.35
HOMA−IR	2.09 (1.15–3.26)	2.47 (1.52–3.55)	1.64 (0.94–2.75)	0.27	0.17–0.35
SBP (mmHg)	109 (100–118)	109 (100–118)	110 (100–117)	−0.01	−0.11–0.09
DBP (mmHg)	65 (59–71)	65 (60–71)	64 (58–71)	0.07	−0.03–0.16
Metabolic syndrome	76 (14.5%)	47 (16.6%)	29 (12.0%)	4.6%	−1.8–11.0%

BMI: body mass index; CI: confidence interval; DBP: diastolic blood pressure; ES: effect size; FFMI: fat-free mass index; FMI: fat mass index; HDL-c: high-density lipoprotein cholesterol; HOMA-IR: homeostatic model assessment insulin resistance; LDL-c: low-density lipoprotein cholesterol; MVPA: moderate-to-vigorous physical activity; PA: physical activity; SBP: systolic blood pressure. Median (Interquartile range), N (%). ES for continuous variables are reported as rank-biserial correlation that ranges from −1 to 1 and indicates the probability that a participant from the second group has a higher value that a participant from the first group. ES for categorial variables was reported as the difference in percentage between groups. MVPA adherence refers to the number of participants who met the recommendations for 60 min/day of MVPA. Full PA adherence refers to the number of participants who met the recommendations for 60 min/day of MVPA and three days of vigorous PA. Promoted and avoided foods scales correspond to the degree of adherence to food consumption recommendations.

**Table 3:** Characteristics, adherence to dietary and physical activity recommendations, and cardiometabolic risk markers by cluster.

				Cluster 1–Cluster 2		Cluster 1–Cluster 3		Cluster 2–Cluster 3	
	Cluster 1	Cluster 2	Cluster 3	ES	95% CI	ES	95% CI	ES	95% CI
*N*	306 (55.7%)	178 (32.4%)	65 (11.8%)						
Age (years)	10.3 (8.3–12.3)	10.5 (8.8–12.1)	11.5 (10.1–13.4)	−0.05	−0.15–0.06	−0.28	−0.42–−0.14	−0.26	−0.40–−0.10
Sex									
Female	165 (53.9%)	106 (59.6%)	25 (38.5%)	−5.6%	−15.0–3.9%	15.0%	1.4–29.0%	21.0%	6.2–36.0%
Male	141 (46.1%)	72 (40.4%)	40 (61.5%)	5.6%	−3.9–15.0%	−15.0%	−29.0–−1.4%	−21.0%	−36.0%–−6.2%
BMI category									
Normal weight	86 (28.1%)	32 (18.0%)	25 (38.5%)	10.0%	2.1–18.0%	−10.0%	−24.0–3.4%	−20.0%	−35.0–−6.3%
Overweight	72 (23.5%)	44 (24.7%)	15 (23.1%)	−1.2%	−9.6–7.2%	0.5%	−11.0–12.0%	1.6%	−11.0–15.0%
Obesity	148 (48.4%)	102 (57.3%)	25 (38.5%)	−8.9%	−19.0–0.7%	9.9%	−4.1–24.0%	19.0%	3.9–34.0%
Physical activity									
MVPA adherence	64 (20.9%)	23 (13.0%)	52 (80.0%)	7.9%	0.7–15.0%	−59.0%	−71.0–−47.0%	−67.0%	−79.0–−55.0%
Full PA adherence	3 (1.0%)	1 (0.6%)	34 (52.3%)	0.4%	−1.6–2.4%	−51.0%	−64.0–−38.0%	−52.0%	−65.0–−38.0%
Moderate PA (min/week)	180 (60–300)	120 (0–300)	240 (120–420)	0.16	0.06–0.27	−0.20	−0.34–0.05	−0.36	−0.49–−0.21
Vigorous PA (min/week)	0 (0–0)	0 (0–0)	300 (240–360)	−0.03	−0.13–0.08	−1.00	−1.00–−1.00	−0.99	−0.99–−0.98
Dietary adherence									
Promoted foods (0–8 scale)	3.47 (2.84–4.25)	4.21 (3.95–4.63)	3.74 (3.32–4.21)	−0.52	−0.59–−0.44	−0.16	−0.31–−0.01	0.42	0.28–0.55
Avoided foods (0–8 scale)	6.42 (6.16–6.68)	7.11 (6.89–7.42)	6.74 (6.42–7.05)	−0.77	−0.81–−0.73	−0.31	−0.44–−0.16	0.45	0.31–0.57
Body mass									
Total fat mass (%)	39.1 (30.0–44.3)	40.7 (35.7–46.4)	36.4 (29.4–42.1)	−0.15	−0.27–−0.02	0.14	−0.03–0.31	0.29	0.12–0.45
Abdominal fat (%)	9.0 (8.0–10.1)	8.7 (7.9–9.8)	9.3 (8.3–15.8)	0.06	−0.09–0.21	−0.18	−0.39–0.05	−0.24	−0.45–−0.01
Total lean mass (%)	59.7 (54.0–69.1)	57.6 (51.4–63.4)	61.9 (56.6–69.6)	0.16	0.04–0.29	−0.15	−0.31–0.03	−0.31	−0.46–−0.14
FFMI (kg/m^2^)	13.0 (11.9–14.4)	13.3 (12.5–14.8)	14.4 (13.4–16.0)	−0.13	−0.25–0.00	−0.40	−0.54–−0.25	−0.31	−0.47–−0.13
FMI (kg/m^2^)	0.9 (0.6–1.1)	1.0 (0.8–1.2)	0.9 (0.6–1.1)	−0.15	−0.28–−0.03	0.03	−0.14–0.20	0.18	0.00–0.35
Cardiometabolic Risk Markers									
Triacylglycerols (mg/dL)	60 (46–83)	63 (47–90)	58 (47–74)	−0.05	−0.16–0.05	0.05	−0.10–0.21	0.10	−0.06–0.26
Cholesterol (mg/dL)	160 (141–179)	165 (147–184)	163 (144–180)	−0.09	−0.19–0.02	−0.06	−0.21–0.10	0.03	−0.13–0.19
HDL−c (mg/dL)	50 (42–61)	48 (40–56)	50 (41–60)	0.13	0.03–0.24	0.01	−0.14–0.17	−0.13	−0.29–0.03
LDL−c (mg/dL)	92 (77–107)	95 (81–116)	95 (83–107)	−0.14	−0.24–−0.03	−0.07	−0.22–0.08	0.06	−0.10–0.22
Insulin (mU/L)	10.1 (5.6–15.5)	11.6 (6.5–17.3)	9.8 (6.8–13.9)	−0.06	−0.17–0.05	0.00	−0.16–0.16	0.08	−0.10–0.24
HOMA−IR	2.01 (1.12–3.26)	2.18 (1.23–3.40)	1.97 (1.24–2.87)	−0.03	−0.15–0.08	0.05	−0.12–0.21	0.09	−0.08–0.27
SBP (mm Hg)	108 (100–118)	109 (101–117)	110 (100–118)	−0.03	−0.14–0.08	−0.07	−0.23–0.08	−0.04	−0.21–0.12
DBP (mm Hg)	65 (60–71)	65 (58–71)	65 (56–70)	0.01	−0.10–0.12	0.07	−0.09–0.22	0.06	−0.10–0.23
Metabolic syndrome	39 (13.5%)	27 (15.9%)	10 (15.4%)	−2.4%	−9.6–4.8%	−1.9%	−12.0–8.7%	0.5%	−10.0–11.0%

Diff: Estimated median difference; CI: Confidence Interval; BMI: body mass index; DBP: diastolic blood pressure; ES: effect size; FFMI: fat-free mass index; FMI: fat mass index; HDL-c: high-density lipoprotein cholesterol; HOMA-IR: homeostatic model assessment insulin resistance; LDL-c: low-density lipoprotein cholesterol; MVPA: moderate-to-vigorous physical activity; PA: physical activity; SBP: systolic blood pressure. Median (Inter quartile range), *N* (%). ES for continuous variables is reported as rank-biserial correlation that ranges from −1 to 1 and indicates the probability that a participant from the second group has a higher value that a participant from the first group. ES for categorial variables was reported as the difference in percentage between groups. Median differences between clusters were estimated using quartile regression with bootstrapped confidence interval.

## Data Availability

The data presented in this study are available on request from the corresponding author. The data are not publicly available due to patient privacy policy.

## Supplementary Materials

The following are available online at https://www.mdpi.com/article/10.3390/nu13092954/s1, Figure S1: Flowchart of the participants’ selection, Table S1: Food frequency questionnaire with the score given by serving for each type of food according to the Spanish Society of Community Nutrition (SENC), Table S2: Characteristics, adherence to dietary and physical activity recommendations, body mass, and cardiometabolic risk markers by BMI category, Table S3: Characteristics, adherence to dietary and physical activity recommendations, body mass, and cardiometabolic risk markers by prevalence or not of metabolic syndrome, Table S4: Characteristics, adherence to dietary recommendations, amount of physical activity, body mass, and cardiometabolic risk markers by adherence to 60 min/day of moderate-to-vigorous physical activity, Table S5: Characteristics, adherence to dietary recommendations, amount of physical activity, body mass, and cardiometabolic risk markers by full adherence to both 60 min/day of moderate-to-vigorous and three days of vigorous physical activity, Supplementary material S1: Statistical methodology of clusters.
