# Supplementary material for: Association of Diet, Physical Activity Guidelines and Cardiometabolic Risk Markers in Children
Source: Nutrients. 2021 Aug 25;13(9):2954. doi: 10.3390/nu13092954 (PMC8466174; doi:10.3390/nu13092954)
Supplement: Supplementary file 1 [file nutrients-13-02954-s001.zip › Supplemental Figure S1.pdf]

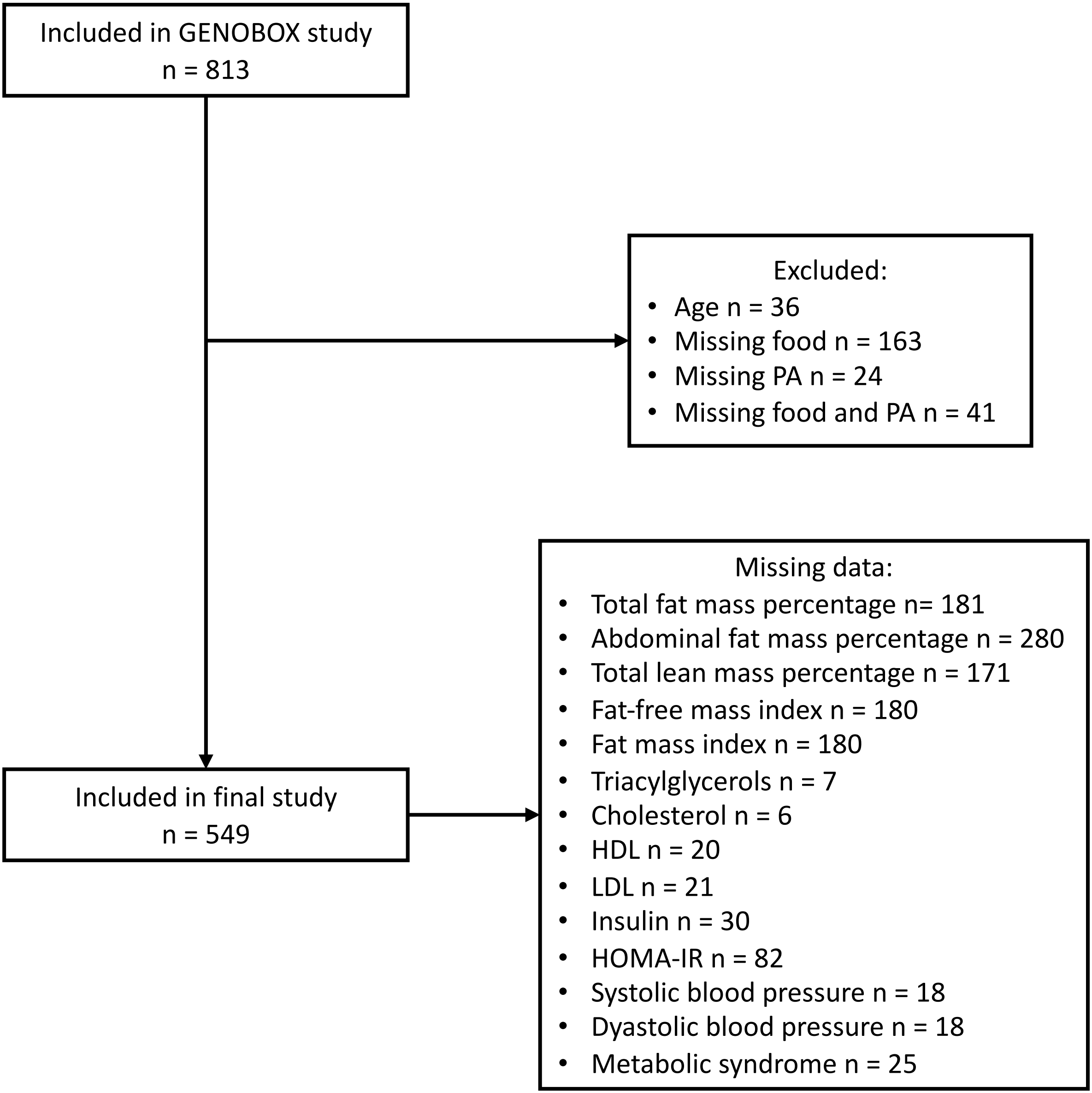
Included in GENOBOX study
n = 813
Excluded:
Age n = 36
Missing food n = 163
Missing PA n = 24
Missing food and PA n = 41
Included in final study
n = 549
Missing data:
Total fat mass percentage n= 181
Abdominal fat mass percentage n = 280
Total lean mass percentage n = 171
Fat-free mass index n = 180
Fat mass index n = 180
Triacylglycerols n = 7
Cholesterol n = 6
HDL n = 20
LDL n = 21
Insulin n = 30
HOMA-IR n = 82
Systolic blood pressure n = 18
Dyastolic blood pressure n = 18
Metabolic syndrome n = 25
