# Supplementary material for: Association of Diet, Physical Activity Guidelines and Cardiometabolic Risk Markers in Children
Source: Nutrients. 2021 Aug 25;13(9):2954. doi: 10.3390/nu13092954 (PMC8466174; doi:10.3390/nu13092954)
Supplement: Supplementary file 1 [file nutrients-13-02954-s001.zip › Supplemental Tables.pdf]

Supplementary Table S1. Food frequency questionnaire with the score given by serving for each type of food according to the Spanish Society of Community Nutrition (SENC).

Food	No servings	Servings per month	Servings per week			Servings per day			
	0	1-3	1	2-4	5-6	1	2-3	4-6	>6
**Water**									
Water	0	1	2	3	4	5	6	7	8
**Dairy**									
Milk	0	3	4	5	6	7	8	2	1
Yogurt	0	3	4	5	6	7	8	2	1
Fresh cheese	0	5	6	7	8	4	3	2	1
**Fruits and vegetables**									
Raw vegetables	0	4	5	6	7	8	3	2	1
Cooked vegetables	0	4	5	6	7	8	3	2	1
Fresh fruit	0	3	4	5	6	7	8	2	1
**Whole grains and potatoes**									
Whole wheat bread	0	3	4	5	6	7	8	2	1
Unsweetened cereals	0	4	5	6	7	8	6	2	1
Whole wheat pasta	0	5	6	7	8	4	3	2	1
Whole wheat rice	0	5	6	7	8	4	3	2	1
Patatoes	0	5	6	7	8	4	3	2	1
**Oil**									
Olive oil	0	3	4	5	6	7	8	2	1
**Meat**									
Cooked beef	0	7	8	6	5	4	3	2	1
Cooked pork	0	7	8	6	5	4	3	2	1
Cooked chicken/turkey	0	6	7	8	5	4	3	2	1
**Eggs**									
Egg	0	6	7	8	5	4	3	2	1
**Fish**									
White fish	0	6	7	8	5	4	3	2	1
Blue fish	0	6	7	8	5	4	3	2	1
**Legumes**									
Lentils	0	7	8	6	5	4	3	2	1
Chickpeas	0	7	8	6	5	4	3	2	1
Beans	0	7	8	6	5	4	3	2	1
**Sweet**									
Sugar	8	7	6	5	4	3	2	1	0
Jam	8	7	6	5	4	3	2	1	0
Cocoa	8	7	6	5	4	3	2	1	0
Nutella	8	7	6	5	4	3	2	1	0
**Sweet snacks**									
Cake, biscuits	8	7	6	5	4	3	2	1	0
Chocolates	8	7	6	5	4	3	2	1	0
Cookies, pastries	8	7	6	5	4	3	2	1	0
Candy	8	7	6	5	4	3	2	1	0
**Salty snacks**									
Nuts	8	7	6	5	4	3	2	1	0
Chips	8	7	6	5	4	3	2	1	0
**Soft drinks**									
Soda	8	7	6	5	4	3	2	1	0
Sport drink	8	7	6	5	4	3	2	1	0
Packaged juice	8	7	6	5	4	3	2	1	0
**Precooked**									
Croquette, small pie...	8	7	6	5	4	3	2	1	0
Pizza	8	7	6	5	4	3	2	1	0
Hamburguer	8	7	6	5	4	3	2	1	0
**Fat and oil**									
Butter	8	7	6	5	4	3	2	1	0
Margarine	8	7	6	5	4	3	2	1	0
Sunflower oil	8	7	6	5	4	3	2	1	0
**Lunchmeat**									
Lunchmeat	8	7	6	5	4	3	2	1	0

Supplementary Table S2. Characteristics, adherence to dietary and physical activity recommendations, body mass, and cardiometabolic risk markers by BMI category.

				NW – OW		NW – OB		OW – OB	
	**Normal weight (NW)**	**Overweight (OW)**	**Obesity (OB)**	**ES**	**95% CI**	**ES**	**95% CI**	**ES**	**95% CI**
N	143 (26.0%)	131 (23.9%)	275 (20.1%)						
Age (years)	10.5 (8.7–12.6)	11.0 (9.6–12.6)	10.5 (8.2–12.2)	-0.11	-0.24 – 0.03	0.04	-0.08 – 0.15	0.15	0.03 – 0.27
Gender									
Female	67 (46.9%)	77 (58.8%)	152 (55.3%)	-12.0%	-24.0% – 0.6%	-8.4%	-19.0% – 2.2%	3.5%	-7.3% – 14.0%
Male	76 (53.1%)	54 (41.2%)	123 (44.7%)	12.0%	-0.6% – 24.0%	8.4%	-2.2% – 19.0%	-3.5%	-14.0% – 7.3%
**Physical activity**									
MVPA adherence	47 (32.9%)	37 (28.5%)	55 (20.0%)	4.4%	-7.3% – 16.0%	13.0%	3.3% – 22.0%	8.5%	-1.2% – 18.0%
Full PA adherence	13 (9.1%)	9 (6.9%)	16 (5.8%)	2.2%	-5.0% – 9.3%	3.3%	-2.7% – 9.3%	1.1%	-4.6% – 6.8%
Moderate PA (min/week)	240 (120–360)	210 (60–360)	120 (0–300)	0.08	-0.06 – 0.21	0.23	0.12 – 0.34	0.13	0.01 – 0.25
Vigorous PA (min/week)	0 (0–60)	0 (0–30)	0 (0–0)	0.02	-0.11 – 0.16	0.08	-0.03 – 0.20	0.06	-0.06 – 0.18
**Dietary adherence**									
Promoted foods (0-8 scale)	3.84 (3.21–4.34)	3.95 (3.28–4.42)	3.89 (3.26–4.42)	-0.07	-0.20 – 0.07	-0.05	-0.17 – 0.06	0.01	-0.11 – 0.13
Avoided foods (0-8 scale)	6.58 (6.26–6.84)	6.84 (6.39–7.13)	6.74 (6.32–7.11)	-0.24	-0.37 – -0.11	-0.17	-0.28 – -0.05	0.07	-0.05 – 0.19
**Body composition**									
Total fat mass (%)	25.2 (20.0–32.0)	39.4 (35.9–42.9)	44.4 (40.5–48.5)	-0.83	-0.87 – -0.77	-0.94	-0.96 – -0.92	-0.45	-0.56 – -0.33
Abdominal fat (%)	8.1 (6.5–15.4)	9.2 (7.9–17.3)	9.0 (8.2–9.8)	-0.21	-0.39 – -0.01	-0.14	-0.31 – 0.05	0.02	-0.13 – 0.18
Total lean mass (%)	74.4 (67.3–80.0)	59.3 (55.2–63.3)	54.0 (49.5–58.4)	0.83	0.77 – 0.87	0.94	0.92 – 0.96	0.42	0.30 – 0.53
FFMI (kg/m^2)	12.4 (11.6–13.8)	13.0 (12.1–14.4)	13.9 (12.9–15.5)	-0.16	-0.31 – -0.01	-0.44	-0.55 – -0.32	-0.31	-0.43 – -0.17
FMI (kg/m^2)	0.4 (0.3–0.6)	0.9 (0.8–1.0)	1.2 (1.0–1.5)	-0.86	-0.90 – -0.81	-0.95	-0.96 – -0.94	-0.59	-0.68 – -0.49
**Cardiometabolic risk markers**									
Triacylglycerols (mg/dl)	52 (41–65)	63 (47–86)	69 (51–95)	-0.28	-0.40 – -0.15	-0.39	-0.49 – -0.29	-0.11	-0.23 – 0.01
Cholesterol (mg/dl)	164 (147–184)	161 (143–182)	162 (140–178)	0.08	-0.06 – 0.21	0.09	-0.03 – 0.20	0.00	-0.12 – 0.12
HDL-c (mg/dl)	60 (51–69)	49 (41–57)	45 (39–53)	0.46	0.35 – 0.57	0.59	0.51 – 0.66	0.16	0.04 – 0.27
LDL-c (mg/dl)	91 (78–106)	95 (79–108)	96 (79–111)	-0.06	-0.20 – 0.08	-0.10	-0.22 – 0.02	-0.04	-0.16 – 0.08
Insulin (mU I/L)	6.8 (4.0–10.8)	10.4 (5.7–14.3)	13.1 (8.7–19.2)	-0.26	-0.39 – -0.13	-0.49	-0.58 – -0.40	-0.26	-0.37 – -0.14
HOMA-IR	1.36 (0.81–2.29)	1.96 (1.11–3.05)	2.68 (1.70–3.91)	-0.24	-0.37 – -0.10	-0.47	-0.56 – -0.37	-0.24	-0.36 – -0.12
SBP (mmHg)	102 (95–109)	107 (100–116)	114 (105–121)	-0.27	-0.39 – -0.14	-0.51	-0.59 – -0.41	-0.26	-0.37 – -0.14
DBP (mmHg)	61 (57–68)	63 (57–69)	68 (62–74)	-0.10	-0.23 – 0.04	-0.40	-0.49 – -0.29	-0.28	-0.39 – -0.16

BMI: body mass index; MVPA: Moderate-to-vigorous physical activity; PA: physical activity; FFMI: fat-free mass index; FMI: fat mass index; HDL-c: high-density lipoprotein cholesterol; LDL-c: low-density lipoprotein cholesterol; HOMA-IR: homeostatic model assessment insulin resistance; SBP: systolic blood pressure; DBP: diastolic blood pressure; ES: effect size; CI: confidence interval.
Median (Inter quartile range), N (%). ES for continuous variables are reported as Rank-Biseral Correlation that ranges from -1 to 1 and indicates the probability that a participant from the second group has a higher value that a participant from the first group. ES for categorial variables was reported as the difference in percentage between groups.

MVPA adherence refers to the number of participants who met the recommendations for 60 min/day of MVPA. Full PA adherence refers to the number of participants who met the recommendations for 60 min/day of MVPA and three days of vigorous PA. Promoted and avoided foods scales correspond to the degree of adherence to food consumption recommendations.

Supplementary Table S3. Characteristics, adherence to dietary and physical activity recommendations, body mass, and cardiometabolic risk markers by prevalence or not of metabolic syndrome.

	No metabolic syndrome	Metabolic syndrome	ES	95% CI
N	174 (69.6%)	76 (30.4%)		
Age (years)	10.6 (8.7–12.3)	11.1 (8.4–12.7)	-0.04	-0.18 – 0.10
Gender[2]				
Female	236 (52.7%)	47 (61.8%)	-9.2%	-22.0% – 3.5%
Male	212 (47.3%)	29 (38.2%)	9.2%	-3.5% – 22.0%
**Physical activity**				
MVPA adherence	118 (26.4%)	19 (25.0%)	1.4%	-9.9% – 13%
Full PA adherence	33 (7.4%)	5 (6.6%)	0.8%	-6.0% – 7.7%
Moderate PA (min/week)	180 (60–300)	120 (0–300)	0.15	0.01 – 0.29
Vigorous PA (min/week)	0 (0–30)	0 (0–15)	0.01	-0.13 – 0.14
**Dietary adherence**				
Promoted foods (0-8 scale)	3.95 (3.26–4.42)	3.68 (3.26–4.32)	0.07	-0.07 – 0.20
Avoided foods (0-8 scale)	6.68 (6.32–7.05)	6.74 (6.37–7.07)	-0.02	-0.15 – 0.12
**Body composition**				
Total fat mass (%)	8.9 (7.8–10.4)	9.1 (8.6–9.9)	-0.59	-0.70 – -0.45
Abdominal fat (%)	60.5 (55.0–69.0)	53.1 (48.6–56.9)	-0.13	-0.33 – 0.08
Total lean mass (%)	13.2 (12.1–14.5)	14.6 (13.1–16.0)	0.57	0.43 – 0.68
FFMI (kg/m^2)	0.9 (0.6–1.1)	1.4 (1.1–1.6)	-0.36	-0.51 – -0.18
FMI (kg/m^2)	8.9 (7.8–10.4)	9.1 (8.6–9.9)	-0.68	-0.77 – -0.56
**Cardiometabolic risk markers**				
Triacylglycerols (mg/dl)	56 (44–75)	96 (65–130)	-0.54	-0.64 – -0.44
Cholesterol (mg/dl)	160 (142–180)	171 (154–184)	-0.16	-0.30 – -0.02
HDL-c (mg/dl)	51 (43–62)	40 (33–48)	0.46	0.34 – 0.56
LDL-c (mg/dl)	92 (78–108)	100 (90–119)	-0.23	-0.36 – -0.09
Insulin (mU I/L)	9.6 (5.3–14.7)	15.7 (10.6–21.9)	-0.46	-0.57 – -0.34
HOMA-IR	1.86 (1.05–3.03)	3.20 (2.15–4.97)	-0.48	-0.59 – -0.35
SBP (mmHg)	106 (100–114)	123 (116–127)	-0.69	-0.75 – -0.61
DBP (mmHg)	64 (58–70)	75 (70–80)	-0.69	-0.76 – -0.61

BMI: body mass index; MVPA: Moderate-to-vigorous physical activity; PA: physical activity; FFMI: fat-free mass index; FMI: fat mass index; HDL-c: high-density lipoprotein cholesterol; LDL-c: low-density lipoprotein cholesterol; HOMA-IR: homeostatic model assessment insulin resistance; SBP: systolic blood pressure; DBP: diastolic blood pressure; ES: effect size; CI: confidence interval.

Median (Inter quartile range), N (%). ES for continuous variables are reported as Rank-Biseral Correlation that ranges from -1 to 1 and indicates the probability that a participant from the second group has a higher value that a participant from the first group. ES for categorial variables was reported as the difference in percentage between groups.

MVPA adherence refers to the number of participants who met the recommendations for 60 min/day of MVPA. Full PA adherence refers to the number of participants who met the recommendations for 60 min/day of MVPA and three days of vigorous PA. Promoted and avoided foods scales correspond to the degree of adherence to food consumption recommendations.

Supplementary Table S4. Characteristics, adherence to dietary recommendations, amount of physical activity, body mass, and cardiometabolic risk markers by adherence to 60 min/day of moderate-to-vigorous physical activity.

	No	Yes	ES	95% CI
N	409 (74.6%)	139 (25.4%)		
Age (years)	10.6 (8.4–12.3)	11.0 (9.1–12.5)	-0.10	-0.21 – 0.01
Gender				
Female	229 (56.0%)	66 (47.5%)	8.5%	-1.6% – 19.0%
Male	180 (44.0%)	73 (52.5%)	-8.5%	-19.0% – 1.6%
BMI category				
Normal weight	96 (23.5%)	47 (33.8%)	-10.0%	-20.0% – -1.0%
Overweight	93 (22.7%)	37 (26.6%)	-3.9%	-13.0% – 5.0%
Obesity	220 (53.8%)	55 (39.6%)	14.0%	4.3%– 24.0%
**Physical activity**				
Moderate PA (min/week)	120 (0–240)	420 (360–600)	-0.86	-0.89 – -0.83
Vigorous PA (min/week)	0 (0–0)	90 (0–255)	-0.51	-0.59 – -0.42
**Dietary adherence**				
Promoted foods (0-8 scale)	3.95 (3.26–4.42)	3.89 (3.26–4.47)	-0.01	-0.12 – 0.10
Avoided foods (0-8 scale)	6.68 (6.32–7.00)	6.74 (6.34–7.11)	-0.06	-0.17 – 0.05
**Body composition**				
Total fat mass (%)	3.95 (3.26–4.42)	3.89 (3.26–4.47)	0.34	0.22 – 0.45
Abdominal fat (%)	6.68 (6.32–7.00)	6.74 (6.34–7.11)	-0.01	-0.19 – 0.16
Total lean mass (%)	3.95 (3.26–4.42)	3.89 (3.26–4.47)	-0.37	-0.47 – -0.25
FFMI (kg/m^2)	6.68 (6.32–7.00)	6.74 (6.34–7.11)	-0.18	-0.30 – -0.05
FMI (kg/m^2)	3.95 (3.26–4.42)	3.89 (3.26–4.47)	0.27	0.15 – 0.39
**Cardiometabolic risk markers**				
Triacylglycerols (mg/dl)	60 (45–86)	62 (49–78)	-0.01	-0.12 – 0.10
Cholesterol (mg/dl)	160 (142–180)	165 (145–184)	-0.07	-0.18 – 0.04
HDL-c (mg/dl)	49 (41–59)	51 (42–62)	-0.09	-0.20 – 0.02
LDL-c (mg/dl)	93 (79–109)	96 (80–107)	-0.03	-0.14 – 0.08
Insulin (mU I/L)	10.9 (5.7–15.8)	9.9 (7.0–14.9)	0.03	-0.09 – 0.14
HOMA-IR	2.14 (1.10–3.28)	1.96 (1.34–3.04)	0.02	-0.10 – 0.13
SBP (mmHg)	109 (100–117)	109 (100–118)	0.01	-0.11 – 0.12
DBP (mmHg)	65 (60–71)	64 (56–70)	0.09	-0.02 – 0.21
Metabolic syndrome	57 (14.8%)	19 (13.9%)	0.9%	-6.4% – 8.2%

BMI: body mass index; PA: physical activity; FFMI: fat-free mass index; FMI: fat mass index; HDL-c: high-density lipoprotein cholesterol; LDL-c: low-density lipoprotein cholesterol; HOMA-IR: homeostatic model assessment insulin resistance; SBP: systolic blood pressure; DBP: diastolic blood pressure; ES: effect size; CI: confidence interval. Median (Inter quartile range), N (%). ES for continuous variables are reported as Rank-Biseral Correlation that ranges from -1 to 1 and indicates the probability that a participant from the second group has a higher value that a participant from the first group. ES for categorial variables was reported as the difference in percentage between groups.

Promoted and avoided foods scales correspond to the degree of adherence to food consumption recommendations.

Supplementary Table S5. Characteristics, adherence to dietary recommendations, amount of physical activity, body mass, and cardiometabolic risk markers by full adherence to both 60 min/day of moderate-to-vigorous and three days of vigorous physical activity.

	No	Yes	ES	95% CI
N	510 (93.1%)	38 (6.9%)		
Age (years)	10.7 (8.6–12.3)	11.3 (9.4–13.1)	-0.16	-0.34 – 0.03
Gender				
Female	279 (54.7%)	16 (42.1%)	13.0%	-5.1% – 30.0%
Male	231 (45.3%)	22 (57.9%)	-13.0%	-30.0% – 5.1%
BMI category				
Normal weight	130 (25.5%)	13 (34.2%)	-8.7%	-26.0% – 8.2%
Overweight	121 (23.7%)	9 (23.7%)	0.0%	-14.0% – 14.0%
Obesity	259 (50.8%)	16 (42.1%)	8.7%	-9.0% – 26.0%
**Physical activity**				
Moderate PA (min/week)	150 (60–300)	330 (240–420)	-0.46	-0.60 – -0.30
Vigorous PA (min/week)	0 (0–0)	308 (240–405)	-0.95	-0.96 – -0.92
**Dietary adherence**				
Promoted foods (0-8 scale)	3.89 (3.26–4.42)	3.79 (3.39–4.25)	0.02	-0.17 – 0.21
Avoided foods (0-8 scale)	6.68 (6.32–7.05)	6.76 (6.49–7.05)	-0.07	-0.26 – 0.12
**Body composition**				
Total fat mass (%)	39.6 (32.4–45.1)	36.4 (29.4–43.0)	0.16	-0.04 – 0.35
Abdominal fat (%)	8.9 (8.0–10.1)	9.7 (8.6–15.4)	-0.22	-0.47 – 0.06
Total lean mass (%)	59.2 (52.9–66.4)	63.0 (55.5–69.9)	-0.19	-0.38 – 0.01
FFMI (kg/m^2)	13.2 (12.2–14.6)	14.6 (13.3–16.2)	-0.35	-0.52 – -0.16
FMI (kg/m^2)	0.9 (0.7–1.2)	1.0 (0.6–1.1)	0.04	-0.16 – 0.24
**Cardiometabolic risk markers**				
Triacylglycerols (mg/dl)	62 (46–86)	52 (46–72)	0.15	-0.04 – 0.33
Cholesterol (mg/dl)	162 (144–181)	161 (140–174)	0.08	-0.11 – 0.27
HDL-c (mg/dl)	49 (41–60)	50 (40–59)	0.00	-0.19 – 0.19
LDL-c (mg/dl)	94 (79–109)	95 (81–104)	0.02	-0.17 – 0.21
Insulin (mU I/L)	10.6 (5.8–15.6)	10.8 (7.7–16.2)	-0.09	-0.27 – 0.11
HOMA-IR	2.11 (1.14–3.26)	2.00 (1.39–2.91)	-0.01	-0.21 – 0.19
SBP (mmHg)	109 (100–118)	110 (100–121)	-0.01	-0.20 – 0.19
DBP (mmHg)	65 (59–71)	64 (52–69)	0.15	-0.04 – 0.35
Metabolic syndrome	71 (14.6%)	5 (13.2%)	-8.7%	-26.0% – 8.2%

BMI: body mass index; PA: physical activity; FFMI: fat-free mass index; FMI: fat mass index; HDL-c: high-density lipoprotein cholesterol; LDL-c: low-density lipoprotein cholesterol; HOMA-IR: homeostatic model assessment insulin resistance; SBP: systolic blood pressure; DBP: diastolic blood pressure; ES: effect size; CI: confidence interval. Median (Inter quartile range), N (%). ES for continuous variables are reported as Rank-Biseral Correlation that ranges from -1 to 1 and indicates the probability that a participant from the second group has a higher value that a participant from the first group. ES for categorial variables was reported as the difference in percentage between groups.

Promoted and avoided foods scales correspond to the degree of adherence to food consumption recommendations.
