# Supplementary material for: Association of Diet, Physical Activity Guidelines and Cardiometabolic Risk Markers in Children
Source: Nutrients. 2021 Aug 25;13(9):2954. doi: 10.3390/nu13092954 (PMC8466174; doi:10.3390/nu13092954)
Supplement: Supplementary file 1 [file nutrients-13-02954-s001.zip › Supplementary material 1.pdf]

Supplementary material 1

In order to cluster the participants based on the amount of physical activity and adherence to dietary guidelines, four variables are used:

1. `advised_foods_z`: Standardized score of degrees of adherence to the recomended intake of foods to be promoted.
2. `advised_against_food_z`: Standardized score of degrees of adherence to the recomended intake of foods to be avoided.
3. `pa_moderate_z`: Standardized values of minutes per week of moderate physical activity.
4. `pa_moderate_z`: Standardized values of minutes per week of vigorous physical activity.

We will perform agglomerative hierarchical clustering using the `agnes` function from the `cluster` package (https://CRAN.R-project.org/package=cluster).

1 Comparing clustering methods

There are several methods available to perform the clustering. Therefore, we first compare the strength of clustering structures produced by different clustering methods using the agglomerative coefficients of the clusters produced by each method. Lower values indicate a tighter, more well-formed cluster.

We compare the following methods: - `average`: Group average method - `single`: Single linkage - `complete`: Complete linkage - `ward`: Ward's method

The agglomerative coefficients of the different methods presented in Table 1 suggest that Ward method produce the strongest cluster structure. We will in continuation, therefore, use Ward's method.

2 Comparing number of clusters

Next step is to determine the number of clusters. To compare different number of clusters (2 to 10), we use the `NbClust` function from the package with the same name (https://CRAN.R-project.org/package=NbClust). The function produces 26 different 26 indices used to determine optimal number of clusters. A detailed discussion of the package and the indices can be found in (Charrad et al. 2014).

Table 2 shows the values of each index for each number of clusters suggested, together with the optimal number of clusters according to each of the indices. The number of clusters that were suggested optimal by most number of indices were 2 (n indices = 9), 5 (n = 7), and 3 (n = 3).

A matrix of bi-variate scatterplots of the variables used to create the clusters, with the cluster belonging of each participant is showed in Figure 1 for 2 clusters, in Figure 2 for 3 clusters, and in Figure 3 for 5 clusters.

Table 1: Agglomerative coefficients for different methods of agglomerative clustering.

Method	Value
average	0.92
single	0.86
complete	0.96
ward	0.98

Table 2: Indices of optimal number of clusters.

	Nr of clusters									
Index	2	3	4	5	6	7	8	9	10	Opt nr
KL	4.11	1.44	0.329	8.88	2.09	19.4	0.364	0.088	1.38	7
CH	132	130	134	146	136	129	123	118	115	5
Hartigan	103	97.1	104	47.4	41.4	37.5	32.5	32.4	28.6	5
CCC	-4.84	-8.08	-8.27	-6.9	-7.05	-7.06	-6.94	-6.91	-6.61	2
Scott	785	1110	1410	1720	1880	2040	2170	2290	2400	3
Marriot	8e+10	9.87e+10	1.02e+11	9.01e+10	9.74e+10	9.99e+10	1.03e+11	1.05e+11	1.06e+11	5
TrCovW	201000	145000	108000	63500	54000	48100	46800	45300	40600	3
TraceW	1760	1480	1260	1050	970	901	843	795	750	5
Friedman	3.24	3.98	4.78	5.66	6.21	6.99	7.58	8.13	8.64	5
Rubin	1.24	1.48	1.74	2.07	2.26	2.43	2.6	2.75	2.92	5
Cindex	0.253	0.256	0.27	0.238	0.227	0.22	0.212	0.206	0.225	9
DB	1.42	1.7	1.43	1.32	1.29	1.44	1.46	1.5	1.44	6
Silhouette	0.377	0.173	0.198	0.205	0.197	0.2	0.136	0.144	0.147	2
Duda	0.808	0.763	0.68	0.668	0.768	0.755	0.725	0.728	0.756	2
Pseudot2	114	94.3	133	66.7	19	56.8	55.3	12.7	35.2	2
Beale	0.571	0.747	1.13	1.19	0.717	0.778	0.908	0.878	0.772	2
Ratkowsky	0.183	0.296	0.315	0.319	0.303	0.288	0.276	0.265	0.256	5
Ball	881	494	314	211	162	129	105	88.4	75	3
Ptbiserial	0.462	0.319	0.419	0.409	0.411	0.423	0.382	0.346	0.351	2
Frey	4.77	-0.357	0.541	0.282	-0.056	1.21	1	-0.0774	0.264	2
McClain	0.161	0.952	0.995	1.7	1.89	1.91	2.57	3.35	3.33	2
Dunn	0.0752	0.0251	0.0294	0.0294	0.0294	0.0294	0.0294	0.0294	0.0326	2
Hubert	0.0014	0.0011	0.0013	0.0014	0.0015	0.0016	0.0017	0.0017	0.0018	0
SDindex	2.61	2.65	2.65	2.41	2.22	2.23	2.51	2.53	2.85	6
Dindex	1.6	1.44	1.36	1.24	1.19	1.16	1.1	1.06	1.04	0
SDbw	1.1	1.41	1.29	1.19	0.96	0.902	0.779	0.729	0.666	10

Opt nr = optimal number of clusters suggested by the index.

The 2 cluster solution almost exclusively cluster participants based on the amount of vigorous PA and does, therefore, does not provide sufficient information to analyze the joint effects of diet and physical activity.

The 3 cluster solution identifies one cluster of higher relative values of advised and advised against foods, with lower relative values of moderate and vigorous PA; one cluster of higher relative vigorous PA (to lesser moderate PA), and average relative levels of advised and advised against foods; and one cluster of lower relative values of advised and advised against foods, with average relative values of moderate and vigorous PA.

The 3 cluster solution provides a more parsimonious solution than the 5 cluster solution, which does not seem to provide enough extra information to defend the added complexity.

We therefore decide that 3 is the optimal number of clusters in the present study.

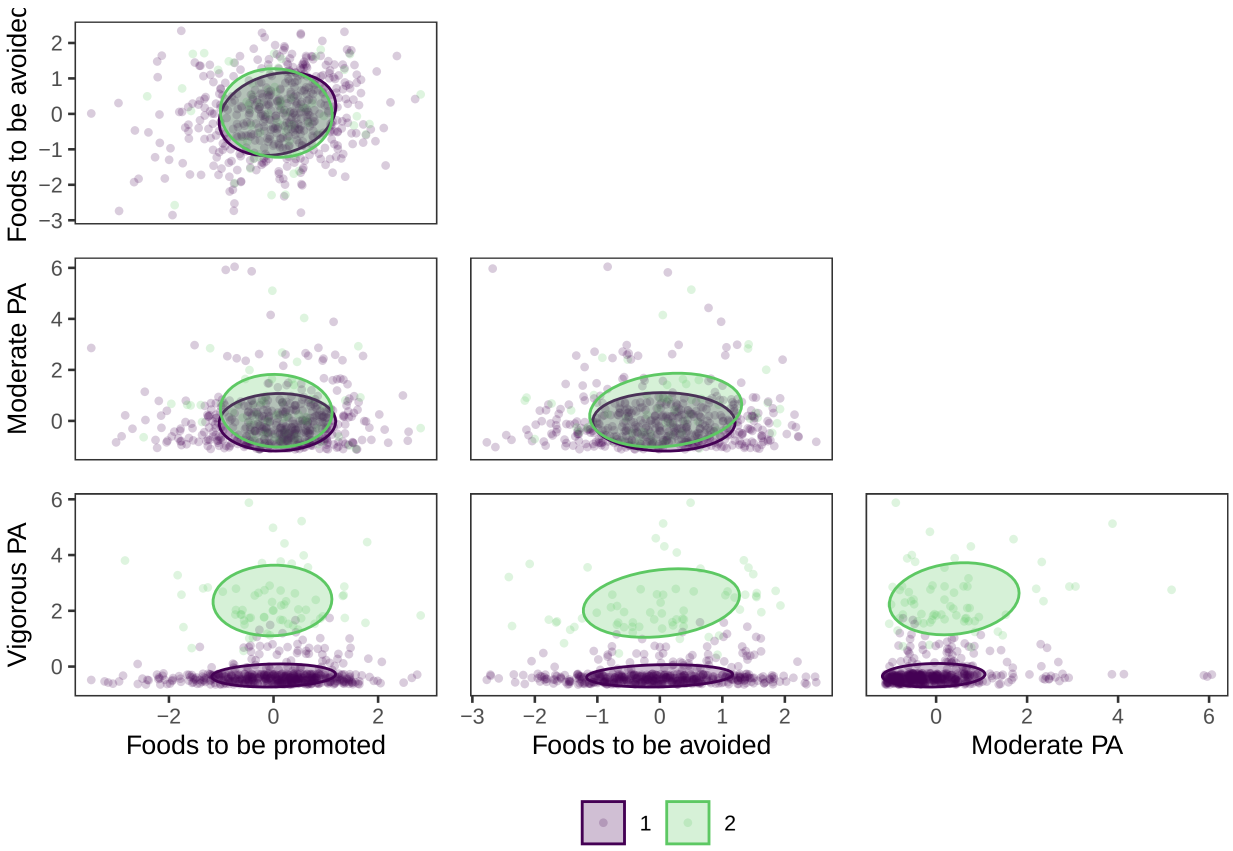

Figure 1: Clustered standardized scores (k = 2) of adherence to dietary recommendations and the amount of physical activity (PA) variables with 50% confidence ellipses

3 Centroids final cluster solution

The centroids of each cluster in the final cluster solution (k = 3) can be seen in Table 3.

4 Heatmap final cluster solution

A heatmap with dendogram of the participants' adherence to dietary recommendation and levels of physical activity can be seen in Figure 4. The dendogram shows the proximity between participants, which are divided into three clusters. The colors indicate standardized levels of adherence to dietary recommendations and levels of physical activity.

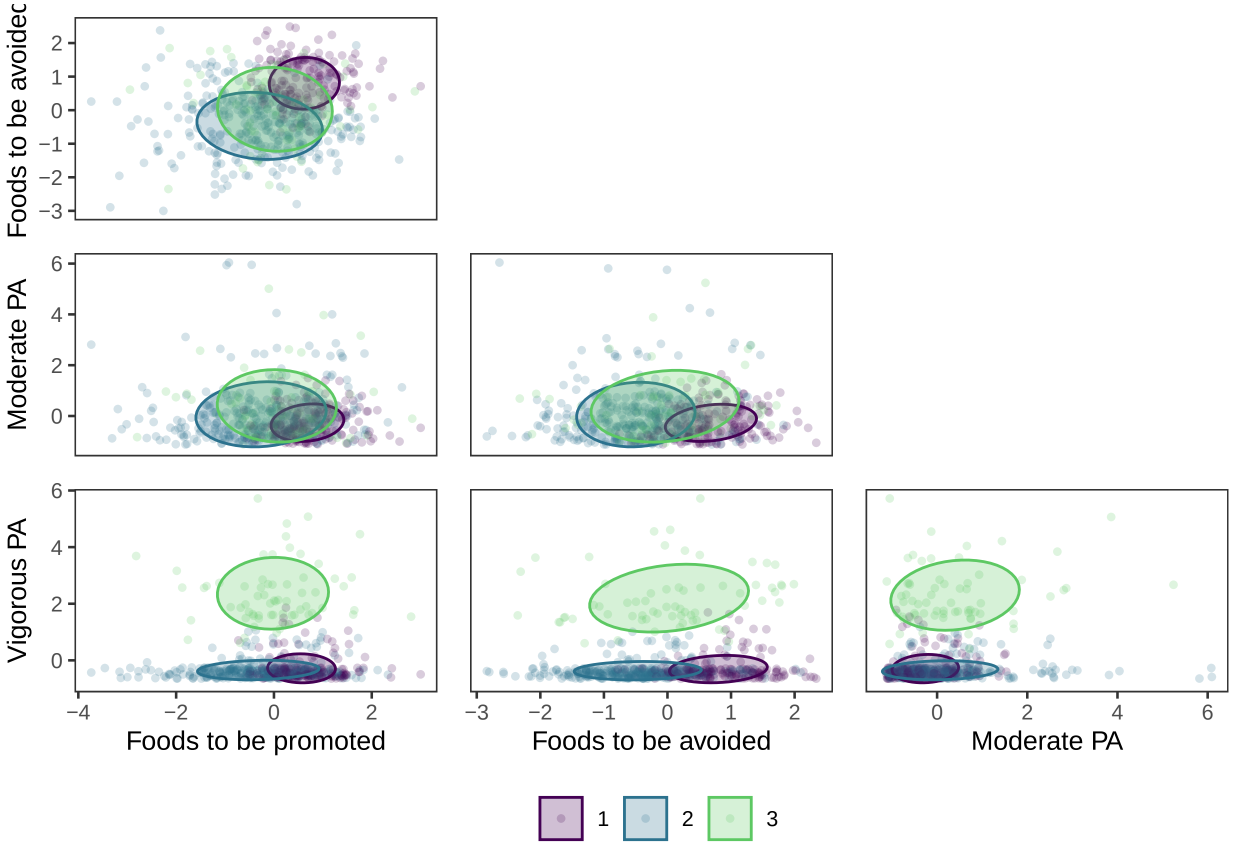

Figure 2: Clustered standardized scores (k = 3) of adherence to dietary recommendations and the amount of physical activity (PA) variables with 50% confidence ellipses

Table 3: Cluster centroids for final cluster solution (k = 3)

Variable	Cluster 1	Cluster 2	Cluster 3
Foods to be promoted z	-0.321	0.563	-0.020
Foods to be avoided z	-0.468	0.800	0.025
Moderate PA z	0.067	-0.262	0.398
Vigorous PA z	-0.341	-0.281	2.370

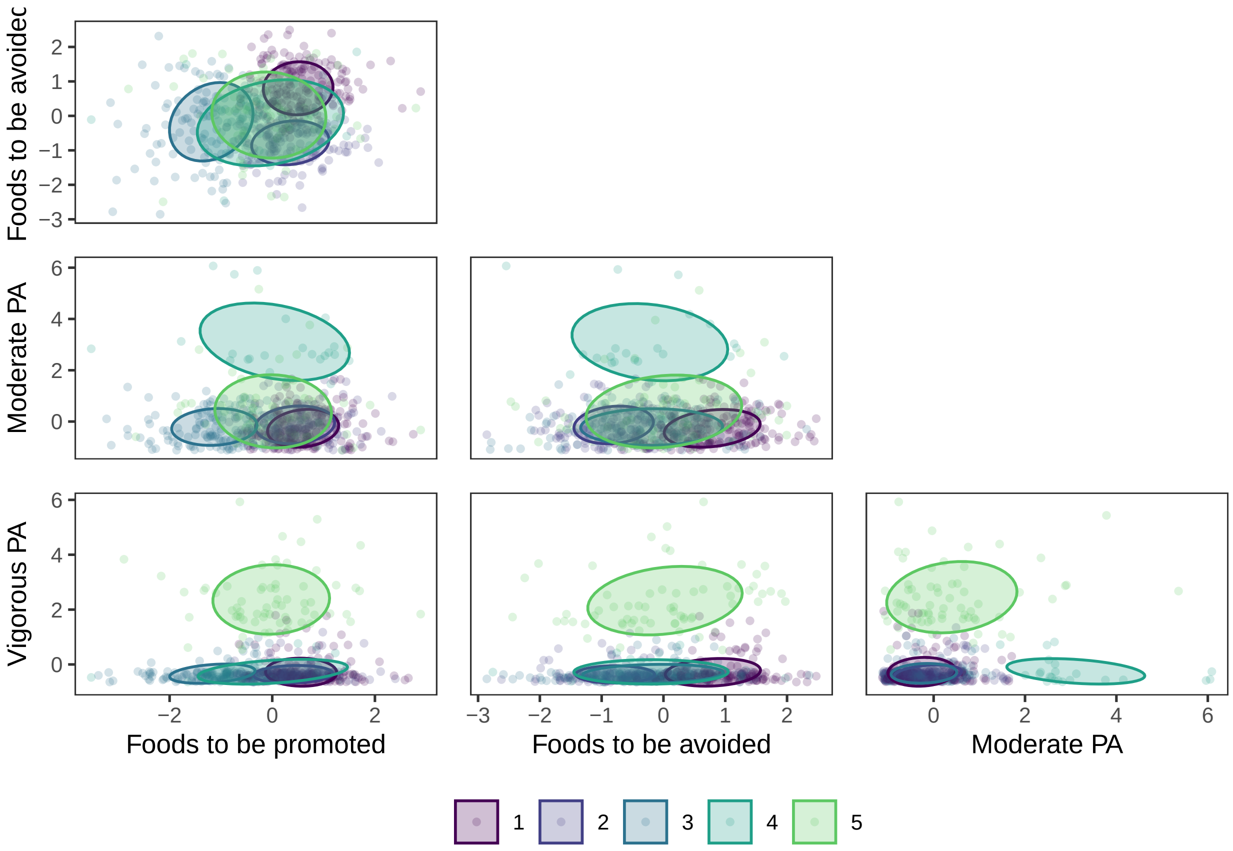

Figure 3: Clustered standardized scores (k = 5) of adherence to dietary recommendations and the amount of physical activity (PA) variables with 50% confidence ellipses

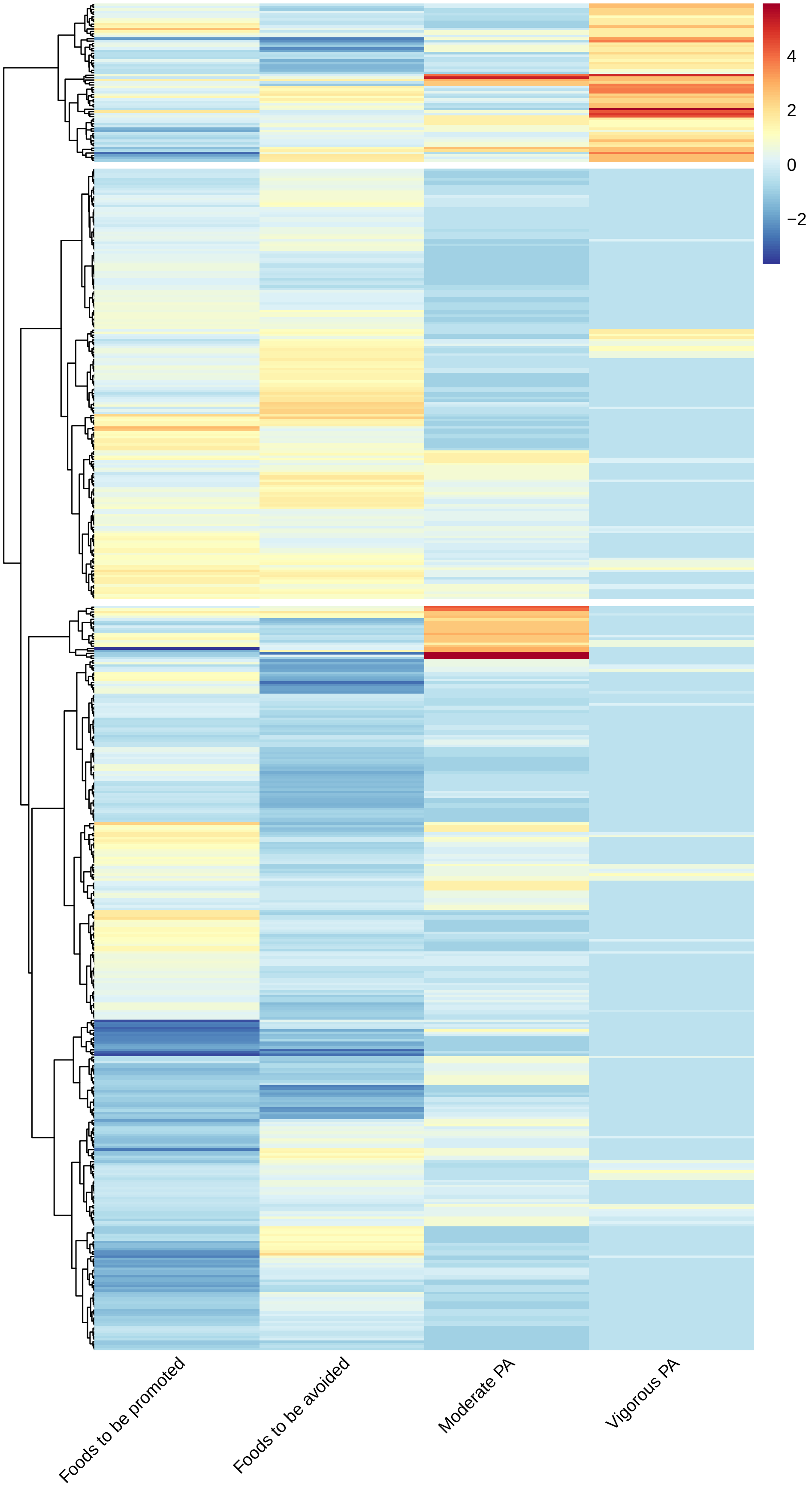

Figure 4: Heatmap of participants' adherence to dietary recommendation and levels of physical activity (PA).

References

Charrad, Malika, Nadia Ghazzali, Véronique Boiteau, and Azam Niknafs. 2014. "NbClust: AnRPackage for Determining the Relevant Number of Clusters in a Data Set." *Journal of Statistical Software* 61 (6). https://doi.org/10.18637/jss.v061.i06.
